# Traumatic Brain Injury as a Potential Risk Factor for Diabetes Mellitus in the Veteran Population

**DOI:** 10.7759/cureus.27296

**Published:** 2022-07-26

**Authors:** Sepehr Saberian, Christian M Mustroph, Fahim Atif, Don Stein, Seema Yousuf

**Affiliations:** 1 Neurological Surgery, Morehouse School of Medicine, Atlanta, USA; 2 Neurological Surgery, Emory University School of Medicine, Atlanta, USA; 3 Emergency Medicine, Brain Research Laboratory, Emory University School of Medicine, Atlanta, USA

**Keywords:** hyperglycemia, chronic traumatic encephalopathy, diabetes mellitus type 2, traumatic brain injury, military trauma, veterans health

## Abstract

This review examines various aspects of traumatic brain injury (TBI) and its potential role as a causative agent for type 2 diabetes mellitus (T2DM) in the veteran population. The pituitary glands and the hypothalamus, both housed in the intracranial space, are the most important structures for the homeostatic regulation of almost every hormone in the human body. As such, TBI not only causes psychological and cognitive impairments but can also disrupt the endocrine system. It is well established that in addition to having a high prevalence of chronic traumatic encephalopathy (CTE), veterans have a very high risk of developing various chronic medical conditions. Unfortunately, there are no measures or prophylactic agents that can have a meaningful impact on this medically complex patient population. In this review, we explore several important factors pertaining to both acute and chronic TBI that can provide additional insight into why veterans tend to develop T2DM later in life. We focus on the unique combination of risk factors in this population not typically found in civilians or other individuals with a non-military background. These include post-traumatic stress disorder, CTE, and environmental factors relating to occupation and lifestyle.

## Introduction and background

Traumatic brain injury (TBI) and its long-term sequelae have become a major area of interest in recent years. Although seen mostly in elite athletes participating in contact sports and military personnel, TBI and its effects in the latter group are of special interest [1]. Warfighters have a significantly higher risk of experiencing TBI and its consequences, attributable to the nature of their work. The majority of TBI diagnoses in this group are a result of blast injuries caused by explosive events. This type of injury can be primary, secondary, tertiary, quaternary, or quinary, depending on the exact mechanism of the explosion, which then can cause the acute and chronic signs and symptoms observed in the affected individual [2]. Not surprisingly, one study reported the incidence of military TBI to be nearly double that of civilian TBI [3]. This is not, however, the only medical condition that is more prevalent in the military population. Observational studies in US veterans have shown that obesity, coronary heart disease, stroke, cancer, chronic obstructive pulmonary disease (COPD), arthritis, mental health disease, renal disease, and type 2 diabetes mellitus (T2DM) are diagnosed more frequently in this group [4]. Although the various etiologies of these conditions have been studied in this population, there is a paucity of data on the mechanisms underlying the relationship between TBI and T2DM in the veteran population.

Diabetes mellitus is a disease of the endocrine system leading to dysregulated glucose homeostasis. Type 1 diabetes mellitus (T1DM) is generally seen in younger individuals, whereas T2DM is seen in older individuals [5]. The precise mechanism for the pathophysiology of the disease is explained in the following section. The National Health and Nutrition Examination Survey (NHANES) reported the prevalence of T2DM in US veterans to be 20.5% in 2013-2014, and the US Department of Veteran Affairs reports a 25% prevalence in this population [6,7]. Interestingly, with a 10% prevalence of T2DM in the general population, the trend is reminiscent of what is observed in the TBI statistics discussed previously [8]. Furthermore, a correlation between elevated blood glucose levels (BGL) on hospital admission in the setting of TBI and mortality has been reported in several studies [9-11]. This review aims to assess potential direct and indirect mechanisms by which T2DM presents in US veterans who have suffered a brain injury during combat.

## Review

Mechanisms and pathophysiology

Traumatic Brain Injury

Although TBI can be broadly categorized as either penetrating or non-penetrating brain injury, non-penetrating injuries are the most common type observed in the civilian population [11]. The mechanism by which non-penetrating TBI occurs involves rapid acceleration or deceleration of the brain in the intracranial space [12]. In the military population, however, the risk of penetrating brain injuries is far greater than in the civilian population due to the high velocity of explosive materials [13]. In both penetrating and non-penetrating TBI, the resulting trauma can be classified into primary and secondary injuries. Primary injury results from a traumatic event that caused physical damage to the brain parenchyma. On the other hand, secondary injury results from various cellular processes that lead to gradual and delayed neuronal damage that can present months to years later in life [14,15]. In the period immediately following the injury, shearing of neurons leads to destabilization of their cell membranes, causing altered membrane potentials, neurotransmitter imbalances, increased glutamate release, as well as other cellular events that cause further damage. Experimental studies have shown that the increase in extracellular potassium levels observed after TBI is a reliable surrogate for measuring neuronal excitotoxicity [16]. Furthermore, the accumulation of calcium ions (Ca+) intracellularly can also lead to further cellular injury by way of mitochondrial damage and induction of oxidative stress [17]. In order to mitigate this overwhelming homeostatic disruption, a large amount of ATP is needed; ATP provides the energy required for restoring levels of these various ions and neurotransmitters [18]. In addition to causing cellular injury, the initial mechanical insult can also lead to shearing of the cerebral vasculature. This can result in hemorrhage, edema, altered cerebral blood flow, vasospasm, coagulopathy, and loss of blood-brain barrier (BBB) integrity [19]. Clinical outcomes of vascular damage can be both mechanical and cellular. Mechanically, accumulation of extravascular blood and edema can compress and injure the adjacent brain parenchyma. Disruption of the BBB can lead to extravasation of various immune cells, including macrophages and lymphocytes, due to a lack of selective permeability. These cells can then release several neurotoxic cytokines such as IL-1β, IL-6, TNF-α, MIP-α, MCP-1, and IL-8, leading to chronic neuroinflammation via direct signaling or indirectly via either direct signaling or indirectly via recruitment of other immune cells into the microenvironment [20,21].

Diabetes Mellitus

Glucose dysregulation is the hallmark of diabetes mellitus. Although two subtypes exist, both are characterized by decreased insulin signaling, resulting in increased BGL. T1DM results from autoimmune-mediated damage to pancreatic β-cells, which are responsible for insulin production and secretion [22]. Therefore, T1DM is a disease of insulin deficiency. In contrast, T2DM is a result of insulin resistance. The pathogenesis of T2DM is associated with several risk factors, including elevated body mass index (BMI), lack of physical exercise, tobacco use, high-glucose and high-fat diets, older age, genetic susceptibility, and medication side effects [8,23]. The most common risk factor, however, is obesity and consumption of high-glucose/high-fat diets [24]. This can lead to overstimulation of the pancreatic β-cells causing the constant release of insulin and subsequent desensitization of insulin receptors. As such, although T2DM patients have normal or elevated insulin levels, their BGL will remain elevated [25]. Over time, overstimulation of the insulin-producing β-cells leads to gradual cellular death, which can further exacerbate the disease [26]. Our review examines various mechanisms by which TBI may lead to hyperglycemia, and specifically T2DM, as long-term sequelae of such injury.

Effects of TBI on the endocrine system

Located within the brain are two critical structures for the proper functioning of the endocrine system: the hypothalamus and the pituitary gland. The hypothalamus is responsible for producing thyrotropin-releasing hormone (TRH), gonadotropin-releasing hormone (GnRH), and growth hormone-releasing hormone (GHRH), corticotropin-releasing hormone (CRH), somatostatin, and dopamine. Following their release into the blood, these hormones reach the anterior pituitary via the hypophysial portal system [27]. Once these hormones bind to their corresponding receptors in the anterior pituitary, they stimulate the production and release of adrenocorticotrophic hormone (ACTH), thyroid-stimulating hormone (TSH), and growth hormone (GH), follicle-stimulating hormone (FSH), and luteinizing hormone (LH) [28]. The only exception to this paradigm is dopamine; at baseline, the constant release of dopamine from the hypothalamus exerts negative regulatory pressure on the pituitary gland, thereby preventing the release of prolactin (PRL) from the anterior pituitary [29]. Contrary to the anterior pituitary, the posterior pituitary does not receive stimulating hormones from the hypothalamus via a portal system. Instead, cell bodies of neurons responsible for producing its hormones, anti-diuretic hormone (ADH) and oxytocin, are located within the hypothalamus with axons extending inferiorly and into the pituitary gland. When a stimulus for the release of these hormones is received, stored vesicles at the terminus of the axons residing in the posterior pituitary fuse with the cell membrane, allowing for these hormones to enter the systemic circulation [30]. All hormones described in this section are paramount for the proper functioning of internal organs during development and appropriate compensatory responses to external stimuli that might disrupt homeostasis.

Given that the pituitary gland and hypothalamus are located intracranially and in proximity to other regions of brain parenchyma, it is not unreasonable to suggest that neurotrauma in the setting of TBI can lead to drastic changes in the endocrine system. Damage to the pituitary can be a result of both physical impact and venous infarction of the hypophyseal portal system [31]. The clinical symptoms observed can vary greatly depending on the extent of the injury as well as the relative deficiencies of specific hormones. GH deficiencies (GHD) tend to be the most common in moderate and severe TBI [32]. Damage to the posterior pituitary can lead to deficiencies in ADH and oxytocin. Low ADH levels can cause diabetes insipidus (DI), a condition in which the renal tubules are unable to reabsorb water. Symptoms of DI include hypernatremia, polyuria, compensatory polydipsia, and hypotonic urine [33]. In a 2012 report, it was shown that six of the 28 veterans studied for hormonal abnormalities following TBI were found to have oxytocin deficiency [34]. The resulting symptoms can include difficulties with social interactions, social bonding, and reduced coping in socially challenging situations [35]. These clinical outcomes resulting from hormonal imbalances can present acutely in a patient experiencing an isolated TBI. In the pediatric population, this imbalance is rarely permanent; in one study, all pituitary hormone levels were found to be normal when blood samples were collected 6.5 ± 3.2 years following the injury [36]. In adults, however, these effects can be either temporary or permanent [24,37].

In the setting of recurrent TBI, chronic traumatic encephalopathy (CTE) can ensue, and affected individuals may have a higher rate of permanent endocrine dysfunction as compared to those patients with isolated TBI. This can be reconciled by two potential explanations: first, repetitive injuries can hinder neurons and other supporting cells from fully recovering. Over time, as these vital cells are not given ample time to adapt to the injury before the next injurious episode, they can become permanently damaged with subsequent cell death. Second, an autoimmune process for the hypopituitarism observed in CTE has been recently proposed. In one study using a rat model, cortical impactions led to the detection of IgG antibodies against both the vascular basal lamina and neuronal components, suggesting the release of hidden neuronal antigens into the systemic circulation, with the body eventually mounting an immune response [38,39]. Interestingly, anti-pituitary antibodies (APA) have also been found to be persistently present for a period of approximately three years in patients who have experienced head trauma [40]. Given these two explanations for the severity of disease in CTE vs TBI, the following section will discuss the implications of CTE as well as the nuanced clinical outcomes observed in the veteran population specifically.

CTE and PTSD in the veteran population: disruption of the endocrine system

In the veteran and military personnel population, CTE is of particular interest. These two groups are at significantly higher risk for developing CTE, compared to the general population, as a result of repetitive and high-impact TBI [41]. Although other individuals, such as professional athletes, have also been shown to be at risk, they do not tend to present with the same medical problems observed in veterans later in life. This may be due to a combination of the individual’s external environment and the extent of the endogenous pathologies caused by CTE. For instance, in professional athletes, 6.5%-25.8% of those who have suffered concussive injuries develop PTSD, whereas in the military population, 34% of individuals who have experienced TBI develop PTSD [42]. Another major difference between the two populations is the nature of their occupation after retirement. Professional athletes are far more likely to hold positions related to the fitness industry than veterans. Additionally, one study reported that 91% of athletes continued practicing physical activity after retirement [43]. On the other hand, retired military personnel are far more likely to lead sedentary lifestyles, with approximately 56% of this population reporting regular physical activity after leaving the military [44]. Although the exact reason for this observation is not clear, potential culprits may include holding more sedentary civilian occupations with long working hours, lack of transportation to fitness facilities, and suffering injuries during active duty that hinder physical exertion as civilians [45]. The combination of these factors (more frequent TBI and CTE, environmental factors, occupational trends) can exacerbate the endocrinological pathologies and clinical outcomes that veterans experience as compared to other individuals with similar traumatic injuries.

PTSD, in addition to causing psychological symptomatology, has also been shown to affect cortisol and catecholamine homeostasis [46]. A hallmark symptom of PTSD is the intense physical reaction and hypervigilance in response to emotional triggers [47]. Although such triggers may not be accompanied by a true physical threat, the emotional perception and physical response to such events are identical to what the individual would experience in combat. As a result, activation of the sympathetic nervous system causes an exaggerated response to otherwise harmless stimuli. As expected, this leads to the release of high amounts of catecholamines, causing classic symptoms of tachycardia, hyperventilation, perspiration, and a subjective feeling of fear [48,49]. Although one would expect cortisol levels to also be elevated in PTSD, its levels tend to be paradoxically lower. This has been attributed to cortisol receptor hypersensitivity [47]. As such, a much lower concentration of the hormone is required for the activation of the receptors. As previously discussed, both catecholamines and cortisol have a strong hyperglycemic effect. Furthermore, the low amount of physical activity observed in the veteran population is another risk factor for high BGL. The combination of such factors leading to higher BGL increases the risk of developing T2DM as compared to individuals who have normal BGL.

Hyperglycemia, diabetes mellitus, and TBI

Several studies have demonstrated the relationship between the severity of TBI and elevated BGL. It has been shown that hyperglycemia in this setting is also associated with higher morbidity, mortality, and longer ICU stays [9,10,50,51]. This section will discuss the major mechanisms by which hyperglycemia occurs following TBI. The most common and well-studied mechanism is hyperglycemia secondary to the body’s acute stress response (ASR). Following any physical injury to the body, a hormonal cascade is initiated to allow for recovery and re-establishment of homeostasis [52]. As discussed in the previous section, injury to the head region specifically can disrupt the hypothalamo-pituitary axis. This combination of the general stress response paired with disruption of the central endocrine modulators results in drastic imbalances in several important hormones. Specifically, the exaggerated increase in catecholamines, cortisol, glucagon, and growth hormone can affect BGL significantly [53-56]. These hormones are strongly related to and act in concert with the sympathetic nervous system. As such, they stimulate glycogenolysis, promote a hypermetabolic environment, increase glucagon secretion, decrease insulin levels, and increase overall insulin resistance [57,58]. Not surprisingly, this constellation of events collectively leads to elevated BGL. Furthermore, an isolated increase in cerebral glycolysis is observed after brain injury, signaling increased cerebral glucose demand. Secondary to this relatively higher oxygen demand and insufficient oxygen supply, cerebral acidosis also ensues due to lactate accumulation by way of anaerobic glycolysis [59]. The second major mechanism promoting hyperglycemia involves various inflammatory processes. Following brain injury, several cytokines, including TNF-α, IL-6, and CD11d, are released into circulation as part of the systemic inflammatory response syndrome (SIRS) [60,61]. The downstream effects of these cytokines include insulin resistance and elevated BGL. SIRS is also associated with elevated cortisol levels, which, combined with the ASR, leads to even higher cortisol release and further propagation of the hyperglycemic state [62]. In some cases, patients with undiagnosed pre-diabetes or sub-clinical hyperglycemia may develop frank T2DM due to the exacerbation of their elevated BGL resulting from the mechanisms outlined earlier [63]. Interestingly, one retrospective study of TBI patients treated in the ICU showed that insulin-treated individuals had better clinical outcomes in the first week of treatment when BGL was maintained in the 5 mmol/L-8 mmol/L range. Conversely, better outcomes were observed when BGL was maintained in the 3.5 mmol/L-6.5 mmol/L range [64]. Although the sample population in this study did not consist solely of combat veterans, these results are still important as they suggest a potentially differential cerebral glucose requirement after TBI as a function of time. In another study, the incidence of T2DM, among other chronic conditions, was measured in two groups: veterans injured in combat and those deployed but not injured. Individuals in the injured veteran group were wounded between 2002 and 2016. The authors found a significant relationship between a higher T2DM diagnosis and having been injured in combat [65]. Of note, although the study does not provide the mechanism of injury for the injured group, one can infer the rate of TBI given that approximately 10%-25% of combat-related injuries are TBI given that the study population was randomly selected [66]. Not surprisingly, a study by Barnes et al. found similar trends toward higher rates of T2DM in the injured group; this study, however, only examined outcomes as a result of TBI suffered during combat as opposed to any kind of combat-related injury as was the case in the previous study [67]. Although these studies were conducted with sufficient statistical power, it is important to recognize the overall lack of literature on the correlation between T2DM and TBI in the military population. Our review has examined the interplay between risk factors associated with TBI in this complex patient population that can lead to T2DM following brain injury. 

**Figure 1:**
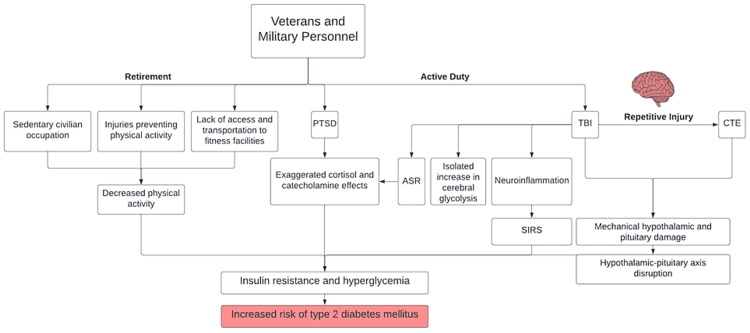
Risk factors for type 2 diabetes mellitus in veterans and military personnel PTSD, post-traumatic stress disorder; TBI, traumatic brain injury; CTE, chronic traumatic encephalopathy; ASR, acute stress response; SIRS, systemic inflammatory response syndrome

In summary, not only is hyperglycemia to be expected after brain injury, but it can also lead to unfavorable outcomes in the affected patient population. Figure 1 summarizes the findings from our review, as stratified by T2DM risk factors for military personnel during active duty and retirement.

## Conclusions

In considering our discussion of the unique combination of risk factors in the veteran population thus far, increased BGL appears to be a common sequala. These risk factors include CTE/TBI, PTSD, and environmental effects such as lifestyle. In the setting of TBI and CTE, the two mechanisms causing hyperglycemia can be classified as mechanical and biochemical. Mechanically, physical damage to the hypothalamus and pituitary glands causes disruptions in the levels of important hormones such as catecholamines, cortisol, GH, and TSH. A common role of these hormones is glucose homeostasis, and as such, deviations from homeostatic levels can lead to a hyperglycemic state. Biochemically, the acute and chronic neuroinflammation and the associated inflammatory cytokines can increase insulin resistance, leading to hyperglycemia. In the setting of PTSD, a condition associated with TBI, the overactivity of the sympathetic nervous system leads to consistently high levels of cortisol and catecholamines, which further propagate the hyperglycemic state. Lastly, the trend toward more sedentary lifestyles in the veteran population is another risk factor for developing hyperglycemia. Taken together, it is feasible to postulate that the combination of these risk factors may play a crucial role in the high rate of T2DM observed in the veteran population as hyperglycemia is a known risk factor for developing diabetes.

Given this complex set of pathologies that accompany brain trauma in the veteran population, a pressing need exists to intensify translational research efforts to mitigate such negative clinical outcomes. These efforts will require both improving animal models of TBI and CTE as well as performing rigorous multicenter preclinical trials. Well-designed clinical trials with relevant clinical characteristics focusing on brain injury, specifically in veterans, are imperative, as this population has a unique physiologic reserve profile that may not be observed in other populations. Further studies on the mechanisms underlying the link between TBI and accelerated T2DM in veterans may lead to improved prophylactic measures with the goal of preventing diabetic sequelae in younger veterans affected by TBI. Thus, many important unresolved questions remain, and we are optimistic that the combined insights from animal models and clinical trials will ultimately provide the knowledge required to create customized multimodal therapies and minimize the burden of disability from TBI in veterans and military personnel.
